# Beware the Ides of Chemotherapy: A Report on Hyperammonemic Encephalopathy Induced by FOLFOX-6 (5-Fluorouracil, Leucovorin, and Oxaliplatin)

**DOI:** 10.7759/cureus.114198

**Published:** 2026-08-09

**Authors:** Sudarsh TS, Sriram Balaji, Dharani K, Krithika S, Karthigeyan TS, Yogesh Subramanian, Aarav J Paul, Sahasyaa Adalarasan, Jayaprakash N, Hariharan C

**Affiliations:** 1 Internal Medicine, Madras Medical College, Chennai, IND; 2 Internal Medicine, Rajiv Gandhi Government Medical College, Chennai, IND; 3 Business Administration, Birla Institute of Technology and Science, Pilani, Pilani, IND; 4 Health Profession Education, Sri Balaji Vidyapeeth University, Pondicherry, IND; 5 Internal Medicine, Rajiv Gandhi Government General Hospital, Chennai, IND; 6 Medicine, Madras Medical College, Chennai, IND

**Keywords:** 5-fluorouracil, colon cancer, folfox-6 regimen, hyperammonemic encephalopathy, oxaliplatin

## Abstract

Hyperammonemic encephalopathy is an uncommon but potentially life-threatening and reversible cause of acute altered sensorium, most often associated with hepatic dysfunction but increasingly recognized in patients receiving chemotherapy. We report the case of a 50-year-old woman who developed progressive disorientation and reduced responsiveness one hour after completing her first cycle of FOLFOX-6 (5-fluorouracil, leucovorin, and oxaliplatin) following hemicolectomy for splenic flexure carcinoma. Initial neuroimaging and infectious workup were unremarkable. Despite correction of transient renal dysfunction, neurological status did not improve. Serum ammonia was markedly elevated, and a diagnosis of non-hepatic hyperammonemic encephalopathy secondary to chemotherapy was made. Prompt discontinuation of FOLFOX and initiation of lactulose resulted in rapid and complete clinical recovery. This case highlights the importance of early recognition of chemotherapy-induced metabolic encephalopathy, particularly fluoropyrimidine-associated toxicity, and emphasizes serum ammonia estimation in unexplained post-chemotherapy neurological deterioration to ensure timely management and favorable outcomes.

## Introduction

Hyperammonemic encephalopathy is a reversible but potentially life-threatening neurotoxic syndrome caused by elevated systemic ammonia levels [1,2]. It is often defined as plasma ammonia ≥50 µmol/L in older children and adults and presents with cerebral dysfunction ranging from irritability and confusion to ataxia, seizures, and cerebral edema. It is most commonly associated with hepatic dysfunction and inborn errors of metabolism, but may also result from infections, porto-systemic shunting, or iatrogenic causes [1,2].

Among chemotherapeutic agents, oxaliplatin is a third-generation platinum compound widely used in gastrointestinal malignancies, particularly colorectal cancer [3]. Compared to cisplatin, it has reduced nephrotoxicity and ototoxicity, and its activity is not dependent on the MMR DNA repair system, contributing to its efficacy in colorectal cancer and lower resistance potential [3,4].

5-Fluorouracil (5-FU) is a pyrimidine analogue commonly used in gastrointestinal cancers [3]. It acts in a cell cycle-specific manner by inhibiting DNA synthesis during the S phase. In combination with oxaliplatin and leucovorin, it forms the FOLFOX regimen, with FOLFOX-6 being the most widely used biweekly outpatient protocol [3].

Despite therapeutic benefits, these agents can cause significant toxicity, including neurological, hematological, hepatic, and hypersensitivity reactions [4]. Rare but serious complications such as posterior reversible encephalopathy syndrome, seizures, hyperammonemic encephalopathy, and delirium have been reported [5-7]. Although the exact mechanism remains unclear, mitochondrial dysfunction and catabolic stress are implicated [5,6], with 5-FU more strongly associated with hyperammonemic encephalopathy than oxaliplatin [5,7].

In the majority of cases of hyperammonemic encephalopathy, liver function abnormalities are present, making liver function tests an important component of the diagnostic evaluation to identify or exclude the underlying cause. However, a few cases, particularly those associated with drug-induced hyperammonemia or Hashimoto's encephalopathy, have been reported in the absence of elevated liver enzymes [8,9].

We report the case of a 50-year-old woman who developed altered sensorium following the initiation of a new chemotherapeutic regimen for splenic flexure carcinoma.

## Case presentation

A 50-year-old woman was transferred from a nearby private hospital with complaints of altered sensorium for the past week. She was apparently normal until family members noticed that she became progressively disoriented, drowsy, and less responsive. There was no history of head trauma or seizures.

**Figure 1:**
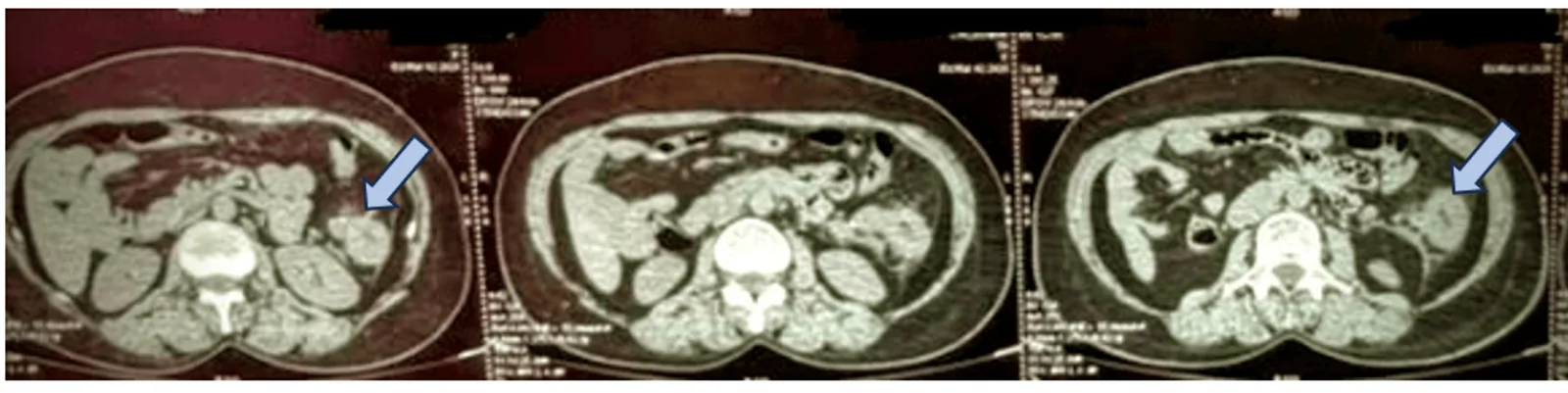
CECT showing colonic wall thickening and fat stranding The figure shows a CECT of the abdomen scan taken prior to surgery, which showed circumferential enhancing wall thickening and fat stranding of the descending colon (indicated by the marked arrows), suggestive of a colonic carcinoma. CECT: contrast-enhanced computed tomography

Her past history was significant. She had initially presented to another tertiary care center with constipation for one month; other history was unremarkable. She is a known case of type 2 diabetes mellitus, hypertension, hypothyroidism, and dyslipidemia and is on regular medications for the same. A contrast-enhanced computed tomography (CECT) of the abdomen showed short-segment circumferential enhancing wall thickening with fat stranding, findings suggestive of a colon carcinoma (Figure 1).

Colonoscopy subsequently confirmed the presence of an ulceroproliferative growth at 45 cm, which obstructed the scope. Initial laboratory findings are given in Table 1. A left hemicolectomy was successfully performed, and the patient was discharged subsequently.

**Table 1:** Baseline laboratory investigations

Parameters	Value	Reference range/impression
White blood cell count (×10^3^/μL)	9.3	4-11
Hemoglobin (g/dL)	11.5	13-17
Platelet count (×10^3^/μL)	272	165-415
Urea (mg/dL)	118	15-40
Creatinine (mg/dL)	1.2	0.59-1.04 (female)
Total bilirubin (mg/dL)	0.6	0.1-1.2
Direct bilirubin (mg/dL)	0.1	0-0.3
Aspartate aminotransferase (U/L)	10	10-40
Alanine aminotransferase (U/L)	13	7-56
Alkaline phosphatase (U/L)	154	44-147
Sodium (mmol/L)	142	135-145
Potassium (mmol/L)	4.4	3.5-5.0
International normalized ratio	1.0	0.8-1.2
Fasting blood glucose (mg/dL)	137	70-99
Thyroid-stimulating hormone (mIU/L)	53.5	0.4-4.0

Two weeks later, the patient was admitted to the same private hospital for chemotherapy as recommended by the tumor board of the surgical center. She received the first cycle of FOLFOX-6, consisting of both oxaliplatin 130 mg and leucovorin 500 mg in 5% dextrose over 48 hours and two hours, respectively, followed by 5-FU 4 g in 1 L normal saline over 46 hours (Table 2). The infusion was initially uneventful; however, about one hour after the end of the infusion, she developed altered sensorium and was transferred to the ICU, where she was managed with intravenous fluids. Due to financial constraints, she later sought care at a tertiary government hospital.

**Table 2:** Chemotherapeutic medications taken by the patient as per the regimen

(Delivery) drug name	Dosage
INJ oxaliplatin	130 mg in 5% dextrose over 24 hours
INJ leucovorin	500 mg in 5% dextrose over 2 hours
INJ 5-fluorouracil	4000 mg in 1000 mL normal saline over 46 hours

**Figure 2:**
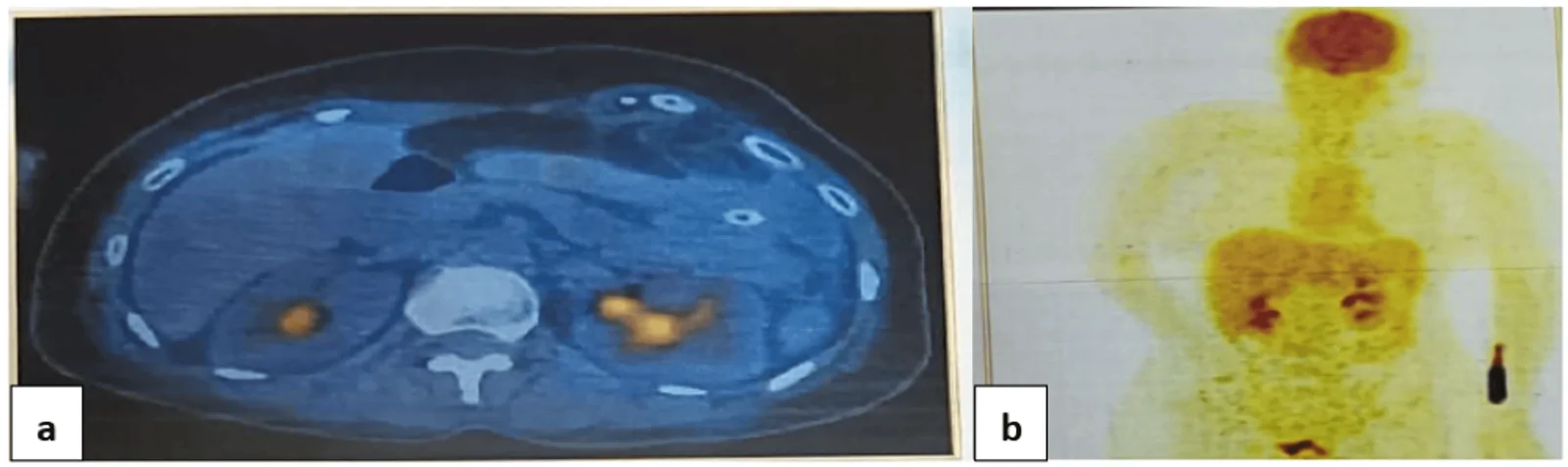
PET-CT scan taken for assessing metastasis The figure shows the PET-CT scan taken for the patient. Image a shows an abdominal section with no significant FDG avidity in the liver, spleen, pancreas, or other organs. Image b shows a full body scan including the brain, where there was no evidence of increased FDG avidity or metabolically active lesions anywhere in the body. PET: positron emission tomography; CT: computed tomography

At presentation, the patient had no additional complaints. Magnetic resonance imaging (MRI) and CT of the brain showed no abnormalities, and a positron emission tomography (PET)-CT scan was advised to evaluate for possible metastasis, but the results of the scan did not support this suspicion (Figure 2).

Liver and renal function tests were performed; renal parameters were markedly elevated with urea at 325 mg/dL and creatinine at 3.6 mg/dL, which was suggestive of pre-renal azotemia secondary to chemotherapy-associated volume depletion, which was completely responsive to intravenous fluid resuscitation. Despite correction with intravenous hydration (3 L normal saline daily), the neurological symptoms in the form of irritability and altered sensorium did not resolve.

Serum ammonia was markedly elevated at 65 μmol/L, consistent with hyperammonemic encephalopathy. In the absence of hepatic dysfunction, infection, or other identifiable causes, the patient was diagnosed with hyperammonemic encephalopathy secondary to the FOLFOX-6 regimen.

The patient was managed with lactulose to reduce serum ammonia levels. Chemotherapy was discontinued, following which the patient improved significantly; regular neurological function was assumed 72 hours after the chemotherapeutic drugs were stopped. On discharge, the tumor board was consulted for further management and consideration of an alternative chemotherapy regimen.

## Discussion

Hyperammonemic encephalopathy is an uncommon but important cause of acute altered mental status, most often associated with hepatic dysfunction but also recognized in multiple non-hepatic causes [1]. It is often underdiagnosed because early presentation overlaps with more common causes of encephalopathy such as sepsis, uremia, and structural intracranial pathology and ammonia is not routinely checked in the initial workup. The underlying mechanism involves impaired ammonia detoxification leading to astrocytic dysfunction, cerebral edema, and global cerebral metabolic failure, while neuroimaging is often normal despite significant clinical deterioration [1,2]. As a result, diagnosis depends heavily on clinical suspicion and early biochemical confirmation.

In the present case, our patient developed progressive encephalopathy within days of receiving first-cycle FOLFOX-6 chemotherapy following hemicolectomy for colon carcinoma. The timing aligns with previously described fluoropyrimidine-associated neurotoxicity, where symptoms typically occur during or shortly after infusion rather than after cumulative exposure. Initial evaluation excluded infective and hepatic causes, and neuroimaging remained unremarkable, consistent with prior reports of chemotherapy-associated metabolic encephalopathy in which imaging is typically non-diagnostic [3].

The diagnostic turning point was the identification of markedly elevated serum ammonia in the absence of hepatic dysfunction. This suggests a "non-hepatic" hyperammonemic picture, increasingly recognized in oncology and critical care but still frequently missed in practice [2,4]. In such cases, neurological impairment is disproportionately severe compared to other laboratory findings, and diagnosis is commonly established only after the exclusion of alternative causes.

When viewed in the context of existing literature, the presentation is highly consistent with fluoropyrimidine-associated hyperammonemic encephalopathy. Reported cases typically describe abrupt neurological deterioration during or shortly after 5-FU administration, elevated ammonia levels, and rapid improvement after drug discontinuation and supportive therapy. Boilève et al. reported similar cases with complete neurological recovery following the withdrawal of 5-FU, highlighting reversibility when recognized early [5]. However, this case is notable given the limited documentation of FOLFOX-6-associated encephalopathy. Oxaliplatin and leucovorin may also contribute, and this case adds to the limited literature describing this association [5,7]. The possible mechanisms in previous literature underlying oxaliplatin's role include mitochondrial dysfunction and transient impairment of hepatic metabolic capacity, leading to reduced ammonia clearance. Leucovorin may also contribute by stabilizing the inhibitory ternary complex between the active metabolite of 5-FU (FdUMP) and thymidylate synthase, thereby potentiating the effects of 5-FU.

Although oxaliplatin was part of the administered FOLFOX regimen, its toxicity profile is primarily peripheral, related to dorsal root ganglion injury and acute sensory neuropathy rather than central metabolic dysfunction. Its mechanism does not directly involve ammonia metabolism or urea cycle disruption [10,11]. In combination therapy reports, fluoropyrimidines are therefore considered the principal agents associated with hyperammonemia, while oxaliplatin is thought to play a minimal or indirect role. However, as mentioned before, oxaliplatin may also act singularly in cases without fluoropyrimidine involvement. This has been demonstrated previously in other tumors, specifically that of the lung and pancreas, where oxaliplatin was postulated to be the main driver of the hyperammonemic encephalopathy seen [12,13].

A notable feature is the occurrence after the first chemotherapy cycle. This aligns with literature suggesting that non-hepatic hyperammonemia is not dose-dependent and may occur unpredictably even after initial exposure. Häberle describes this as a threshold phenomenon influenced by individual metabolic reserve rather than cumulative toxicity, which may explain early onset in some patients [2].

The transient renal dysfunction may have contributed as a secondary factor by reducing ammonia clearance, although it is unlikely to be the primary cause. Non-hepatic hyperammonemia is increasingly understood as multifactorial, where metabolic stressors such as dehydration, catabolic state, and organ dysfunction collectively overwhelm ammonia elimination pathways [4]. In this setting, chemotherapy likely acted as the primary precipitant.

The most clinically important aspect of this case is the rapid and complete neurological recovery following the discontinuation of chemotherapy and the administration of lactulose. Lactulose reduces intestinal ammonia production and enhances nitrogen elimination, forming the basis of standard therapy [6]. Recovery within days is consistent with previously reported chemotherapy-associated hyperammonemic encephalopathy, where improvement typically occurs within 24-72 hours after intervention [1,5,6]. 

The major limitation in this particular case was the absence of gene testing done for dihydropyrimidine dehydrogenase (DPD), which is responsible for breaking down 5-FU. This test is usually done to confirm a possible genetic predisposition to 5-FU toxicity in such patients, due to the deficiency of this enzyme [14]. While this was not done to confirm the etiology of this case, once the patient improved on the cessation of the drug, it proved redundant to perform such a test apart from that for academic purposes.

## Conclusions

Hyperammonemic encephalopathy is a rare but reversible neurotoxic complication that may occur in patients receiving fluoropyrimidine-based chemotherapy, particularly FOLFOX regimens. This case highlights the importance of considering non-hepatic causes of acute altered mental status in oncology patients when structural, infectious, and metabolic etiologies are excluded.

Measurement of serum ammonia provided the key diagnostic clue and guided the timely discontinuation of the offending agents, leading to complete neurological recovery. Although 5-FU remains the most likely precipitant, contributory roles of combination therapy and transient renal dysfunction cannot be excluded. Increased clinical awareness is essential to ensure rapid recognition, prevent morbidity, and reduce mortality.
